# Brain transcriptomic, metabolic and mitohormesis properties associated with *N*-propargylglycine treatment: A prevention strategy against neurodegeneration

**DOI:** 10.1016/j.brainres.2023.148733

**Published:** 2023-12-20

**Authors:** Fadzai Teramayi, Joanna Bons, Madeleine Scott, Gary K. Scott, Ashley Loureiro, Alejandro Lopez-Ramirez, Birgit Schilling, Lisa M. Ellerby, Christopher C. Benz

**Affiliations:** a Buck Institute for Research on Aging, Novato, CA, USA; b Center for Biomedical Informatics, Department of Medicine, Stanford University School of Medicine, Stanford, CA, USA

**Keywords:** Proline dehydrogenase (PRODH), *N*-Propargylglycine (*N*-PPG), Brain mitohormesis, Neurodegeneration

## Abstract

There is an urgent need for new or repurposed therapeutics that protect against or significantly delay the clinical progression of neurodegenerative diseases, such as Huntington’s disease (HD), Parkinson’s disease and Alzheimer’s disease. In particular, preclinical studies are needed for well tolerated and brain-penetrating small molecules capable of mitigating the proteotoxic mitochondrial processes that are hallmarks of these diseases. We identified a unique suicide inhibitor of mitochondrial proline dehydrogenase (Prodh), N-propargylglycine (*N*-PPG), which has anticancer and brain-enhancing mitohormesis properties, and we hypothesize that induction of mitohormesis by *N*-PPG protects against neurodegenerative diseases. We carried out a series of mouse studies designed to: i) compare brain and metabolic responses while on oral *N*-PPG treatment (50 mg/kg, 9–14 days) of B6CBA wildtype (WT) and short-lived transgenic R6/2 (HD) mice; and ii) evaluate potential brain and systemwide stress rebound responses in WT mice 2 months after cessation of extended mitohormesis induction by well-tolerated higher doses of *N*-PPG (100–200 mg/kg x 60 days). WT and HD mice showed comparable global evidence of *N*-PPG induced brain mitohormesis characterized by Prodh protein decay and increased mitochondrial expression of chaperone and Yme1l1 protease proteins. Interestingly, transcriptional analysis (RNAseq) showed partial normalization of HD whole brain transcriptomes toward those of WT mice. Comprehensive metabolomic profiles performed on control and *N*-PPG treated blood, brain, and kidney samples revealed expected *N*-PPG-induced tissue increases in proline levels in both WT and HD mice, accompanied by surprising parallel increases in hydroxyproline and sarcosine. Two months after cessation of the higher dose *N*-PPG stress treatments, WT mouse brains showed robust rebound increases in Prodh protein levels and mitochondrial transcriptome responses, as well as altered profiles of blood amino acid-related metabolites. Our HD and WT mouse preclinical findings point to the brain penetrating and mitohormesis-inducing potential of the drug candidate, *N*-PPG, and provide new rationale and application insights supporting its further preclinical testing in various models of neurodegenerative diseases characterized by loss of mitochondrial proteostasis.

## 1. Introduction

Neurodegenerative diseases lack effective therapeutics to prevent or correct any of their underlying hallmark features including aberrant proteostasis, pathological protein aggregation and impaired mitochondrial homeostasis (Wilson et al., 2023). Despite remarkable progress and a rich body of research on causative mutations that drive the growing class of neurogenetic diseases known as repeat expansion disorders exemplified by Huntington’s disease (HD), only symptom-palliating medications are available for clinical use today (Ellerby, 2019).

HD remains the best studied of several clinically distinct polyglutamine (polyQ) repeat diseases. Many mouse models are available for researchers to explore the phenotypic and pathogenetic variations determined by how those models were designed, including the polyQ repeat lengths in their mutant Huntingtin (mHTT) proteins and their pan-neuronal or cell (neuronal or non-neuronal)-specific mHTT expression patterns (Creus-Muncunill and Ehrlich, 2019). In almost all the models, brain cells accumulate aggregates of intracellular mHTT along with aberrant protein localization in their mitochondria associated with altered mitochondrial structure, loss of mitochondrial dynamics and proteostatic quality control functions (Creus-Muncunill and Ehrlich, 2019). These observations have led many investigators to surmise that altered proteostasis and mitochondrial stress directly contribute to loss of neuronal viability and synaptic network dysfunction in both mouse models and HD patients (Hu et al., 2021; Machiela et al., 2020; Singh and Agrawal, 2022; Wilson et al., 2023).

The oldest and still widely used HD model is the R6 transgenic mouse. The pan-cellular expressing R6/1 and R6/2 sublines each express 67 amino acids (encoded by *HTT* exon-1) of the N-terminal human mHTT protein driven by the human *HTT* promoter. However, they differ in the number of CAG repeats: ~115 for R6/1 (Mangiarini et al., 1996) and ~ 150 for R6/2 (Davies et al., 1997; Li et al., 2005). Both sublines exhibit subcellular mitochondrial abnormalities (Creus-Muncunill and Ehrlich, 2019; Petersen et al., 2022) and develop weight loss, enlarged cerebral ventricles, neuronal atrophy, progressive increase of mHTT aggregates, motor alterations and cognitive deficits but at vastly different rates. R6/2 mice are the most aggressively progressing of all HD models: they develop symptoms as early as 6 weeks, show a wider scope of mHTT brain inclusions and rapidly die within 16 weeks of birth (Li et al., 2005). Thus this model represents a worst case *in vivo* disease scenario but is not a genetically accurate mouse model of HD.

Notably, investigators using a different pan-cellular HD mouse model expressing full-length mHTT with 128 CAG repeats (YAC128) recently reported a critical link between mHTT-induced neurodegeneration and mitochondrial targeting of a transcription factor (HSF1) that sustains mitochondrial proteostasis and induces the mitochondrial unfolded protein response (UPR^mt^) (Liu et al., 2022). This supports the decade old observation that the polyQ-expanded HTT protein binds to mitochondria and triggers an UPR^mt^ (Costa and Scorrano, 2012; Wang et al., 2022). Additional observations of altered mitochondrial proteostasis in mouse models and patients with even more prevalent neurodegenerative diseases have experts now calling for an entirely new therapeutic strategy that bolsters mitochondrial fitness to treat a broad array of related neuroprotinopathies (Lautenschlager et al., 2020; Hu et al., 2021; Singh and Agrawal, 2022).

Enhancing central nervous system (CNS) mitohormesis (ie., stressing CNS mitochondria to induce a positive adaptive proteostatic response) has been suggested as a novel HD treatment approach (Fu et al., 2019; Stoker et al., 2022). However, until very recently no systemically well-tolerated, mitochondria-targeted, brain-penetrating, and mitohormesis-inducing drug candidate has been proposed and tested *in vivo*. In pursuing *N*-propargylglycine (*N*-PPG) as a unique suicide inhibitor of mitochondrial proline dehydrogenase (Prodh) and potential anticancer therapeutic, we observed that, in both human cancer cells and normal mouse tissues, its irreversible inhibition and structural distortion of Prodh protein induces UPR^mt^ and thereby activates mitohormesis, a property independent of *N*-PPG’s anticancer activity but consistent with its lack of any obvious host toxicity when administered orally for 9 days at 50 mg/kg (Scott et al., 2019; Scott et al., 2021).

We sought to determine if the potential brain mitohormesis-inducing properties of *N*-PPG warrant its repurposing as a novel therapeutic to prevent or mitigate the proteotoxic mechanisms driving neurodegenerative diseases. Here we report a series of preclinical mouse studies detailing and comparing mouse tolerability and behavioral effects as well as brain protein, RNA and metabolite responses in wildtype (WT, B6CBA) and short-lived R6/2 HD mice receiving mitohormesis-inducing doses of *N*-PPG. We looked also for evidence of a sustained rebound response in the brains of WT mice completing an extended course of mitohormesis-induction with higher and longer *N*-PPG dosing.

## 2. Materials and methods

### 2.1. Study drug and antibodies

*N*-Propargylglycine (*N*-PPG; #58160–95–5) was purchased from BOC Sciences (Shirley, NY). Antibodies used in this study included mouse monoclonals against β-actin (C4 sc-47778), Prodh (A-11 sc-376401), Dmgdh (E6 sc-393178, Gnmt (A-4 sc-166834), and Rieske FeS IgG (A-5 sc-271609) from Santa Cruz Biotechnology (Santa Cruz, CA); Yme1l1 (#11510–1-AP), Grp78 (#11587–1-AP), and Sardh (#22762–1-AP) rabbit polyclonals from ProteinTech^™^ (Rosemont, IL); Prodh2/Hypdh rabbit polyclonal (PA5–62366) from Thermo-Fisher (Ashville, NC); HRP-conjugated goat anti-mouse secondary (#1706516) from BioRad Laboratories, Inc. (Hercules, CA); and HRP-conjugated mouse anti-rabbit monoclonal (#211–032–171) from Jackson ImmunoResearch Laboratories (West Grove, PA).

### 2.2. Vehicle and N-PPG treated WT and R6/2 mice

The animal facility at the Buck Institute for Research on Aging is accredited by AAALAC International (Unit Number 001070). All protocols and procedures described herein were approved by the Buck’s Institutional Animal Use Committee. Male R6/2 and B6CBA mice were purchased from the Jackson Laboratory (Bar Harbor, ME) and were received at ~ 4 weeks of age. CAG repeat length was confirmed by PCR. Three sequential studies were performed to compare *N*-PPG treatment effects against those of saline vehicle (Veh) treated wildtype (WT) or R6/2 (HD) mice, as schematically illustrated herein. Commercially obtained *N-*PPG (150 mg) was dissolved in 1 mL of 0.9 % saline and the solution was neutralized to pH 6.9 with 1 M NaOH to achieve final solution volumes of *N*-PPG. According to each of the three study protocols, indicated doses of *N*-PPG (50, 100, and 200 mg/kg) were administered daily to each mouse by oral gavage, with Veh-treated control mice similarly gavaged with 200 μL of saline. Mice were weighed periodically as indicated, and all drug doses were adjusted according to mouse weight. From 1 to 4 h after their final oral gavage treatment mice were anesthetized with isofluorane (Butler Schein) and cervically dislocated, and mouse tissues (brain, kidney, and/or blood) were immediately removed and snap frozen with dry ice.

### 2.3. Tissue extractions

Blood was collected in EDTA tubes and then stored at −70 °C. Snap frozen tissues were pulverized completely under liquid nitrogen, with separate frozen aliquots of the pulverized samples designated for RNA extraction, protein lysates, or metabolomic analyses.

### 2.4. Immunoblotting

Protein lysates from frozen aliquots were first sonicated in low salt buffer supplemented with detergent (10 mM Tris pH 7.5, 50 mM NaCl, 1 % SDS). Prior to immunblotting, protein content was determined by Bradford Coomassie Assay (BCA) kit (Pierce, Rockford, IL) and lysates diluted into 2X Laemmli sample buffer. Immunoblotting was performed as described (Scott et al., 2019; Scott et al., 2021) using polyvinylidene fluoride (PVDF) membranes blocked with 5 % non-fat milk in TBST (Tris-buffered saline with 0.1 % Tween-20) incubated with primary and then secondary antibodies conjugated to horse radish peroxidase (HRP), and the resulting immunoblot signals were scanned for densitometry.

### 2.5. RNA sequencing

For total RNA extraction and RNA sequencing, frozen tissue samples were pulverized further in Trizol (Thermo Fisher, Ashville, NC) and then resuspended into Trizol with sonication. The resulting Trizol solution was processed according to the manufacture’s protocol with RNA yields of at least 100 μg of RNA, from which at least 12 μg/sample was shipped as instructed on dry ice to Gene-Wiz (Azenta Life Sciences, South Plainfield, NJ) for full transcriptome sequencing (RNAseq). As described (Scott et al., 2021), raw and normalized log2-scaled gene expression values (transcripts per million) were reported back. The Genewiz bioinformatics workflow used Trimmomatic (v.0.36) to remove poor quality sequences (Bolger et al., 2014), and trimmed reads were then aligned to the *Mus musculus* GRCm38 (mm10) reference genome using STAR (v.2.5.2b) (Dobin et al., 2013). The resulting BAM files were input into featureCounts from Subread (v.1.5.2) to produce the final gene count matrix (Liao et al., 2013). DESeq2 (v. 1.26.0) was used to normalize gene counts and perform all differential expression analyses (Love et al., 2014). Differentially expressed genes were defined as those with a rounded p-value less than or equal to 0.05. All primary RNAseq data returned from GeneWiz, analyzed and reported in this study, have been deposited into GEO for public access (GSE252068).

### 2.6. Metabolomic analysis

For targeted metabolomic analysis, aliquots of at least 50 mg from each frozen (−80 °C) tissue sample, in addition to at least 200 μL of blood collected in EDTA tubes, were transferred into soft tissue homogenizing CK14 tubes (Bertin Corp, Rockville, MD) and shipped overnight on dry ice to the Duke University School of Medicine’s Proteomics and Metabolomics Core Facility at the Center for Genomics and Computational Biology (Durham, NC).

#### 2.6.1. Brain and kidney tissue homogenization

Based on provided weights, 10 μL of homogenization buffer (30 % 1:1 methanol:water, 70 % 3:1 methanol:chloroform) was added to each tube for every mg of tissue. Then, samples were homogenized using three 10-second pulses in the Precellys Evolution system between which samples were chilled using the Cryolys cooling system. The samples were stored frozen at −80 °C until ready for further preparation.

#### 2.6.2. Sample preparation using the biocrates MxP Quant 500 Kit

Mouse brain, kidney, and blood samples were prepared using the MxP Quant 500 Panel kit (Biocrates Life Sciences AG, Innsbruck, Austria) in strict accordance with their detailed protocol. Kits were supplied with internal standard applied to each well of the 96-well extraction plate. Ten μL of each blank, calibration standard, Biocrates QC, Global Reference QC were added to the appropriate wells. Thirty μL of each sample and sample pool quality control (SPQC) were added to the appropriate well. The plate was then dried under a gentle stream of nitrogen for 30 min. Samples were derivatized with phenyl isothiocyanate, then eluted with 5 mM ammonium acetate in methanol. Samples were diluted with either 80:20 water:methanol for the ultra-performance liquid chromatography (UPLC)-tandem mass spectrometry (MS/MS) analysis (1:1) or running solvent (Biocrates AG) for flow injection analysis (FIA)-MS/MS (20:1).

#### 2.6.3. Mass spectrometric analysis

Ultra-High-Performance LC (UHPLC) separation was performed using an Exion AD liquid chromatograph (Sciex, Framingham, MA) with a Waters Acquity 2.1 mm x 50 mm 1.7 μm BEH C_18_ column fitted with a Waters Acquity BEH C_18_ 1.7 μm Vanguard guard column with a stainless-steel pre-column mixer (Biocrates AG). Analytes were separated using a gradient from 0.2 % formic acid in water, to 0.2 % formic acid in acetonitrile. Total UPLC analysis time was approximately 6 min per sample. For UPLC-MS/MS measurements, samples were directly injected into a QTRAP 6500+ (Sciex) operating in Multiple Reaction Monitoring (MRM) mode and analyzed using two methods, one in Electrospray Ionization Positive mode (ESI + ) and one in negative mode (ESI-). For each analyte and internal standard, MRM transitions were collected over the appropriate retention time. For flow injection ion spray MS/MS (FIA-MS/MS) measurements, samples were analyzed using two separate methods with a total analysis time of approximately 3.8 min per injection using ESI + and MRM mode on a Xevo TQ-S triple quadrupole mass spectrometer (Waters). Sample injection was performed with an ACQUITY UPLC (Waters). For each analyte and internal standard, compound-specific precursor to product ion transitions were collected.

#### 2.6.4. MS data processing

The UPLC-MS/MS data were imported into Skyline-daily (version 21.1.1.9.335, https://skyline.ms/) for peak integration, calibration, and concentration calculations (Adams et al., 2020). Export from Skyline was processed using a custom visual basic macro for importing into MetIDQ software (Biocrates AG). The FIA-MS/MS data for acylcarnitines, monosaccharides (hexose), diglycerides, triglycerides, lysophosphatidylcholines, phosphatidylcholines, sphingomyelins, ceramides, and cholesteryl esters were analyzed using MetIDQ software. Data were adjusted for sample dilution factors. To ensure robust results, metabolites with > 50 % missing values or values below LOD (level of detection) and metabolites with coefficient of variation > 30 % on technical triplicate of the corresponding SPQC were excluded. Remaining missing values were imputed by half of LOD of each analyte (Suppl. Table S3A-C, S4A, S5A-C). Metabolomic analysis was performed on HD mice treated with either *N*-PPG or Veh. Although over 260 metabolites representing 19 different compound groups were commonly detected in our mouse brain, kidney and blood samples, our intended study focused only on the subset of 20 amino acids and 19 amino acid-related metabolites (Suppl. Figure S4; Suppl. Table S3A-C). The metabolite concentration values were Log10 transformed and further analyzed using MetaboAnalyst 5.0 v5.0 (Pang et al., 2021). Differences in metabolite concentrations in each tissue were evaluated using two-sample t-tests, followed by p-value correction for multiple testing, or one-way ANOVA analysis and Fisher’s LSD post-hoc testing. A q-value or false discovery rate (FDR) < 0.05 was applied to consider statistical significance in any of the analyses (Suppl. Table S3D-F, S4B, S5D-F). Hierarchical cluster heatmaps and boxplots were created using MetaboAnalyst 5.0.

### 2.7. Mouse metabolic cage measurements

WT-B6CBA and HD-R6/2 male mice were placed into metabolic cages to assess oxygen consumption, carbon dioxide production, energy expenditure, food and water consumption measured by indirect calorimetry. The indirect calorimeter is a ventilated, open-circuit system, through which a flow of fresh air is passed (Tschop et al., 2011); this system collects and mixes the expired air, measures the flow rate and analyzes the gas concentration of the incoming and outgoing air for both oxygen and carbon dioxide. All forms of mouse activity including wheel running are measured by infrared counts of animal movement (Killion et al., 2018).

Measurements of energy expenditure in mice were calculated based on the amount of oxygen consumed and carbon dioxide produced. Parameters were recorded during light and dark cycles, each 12 h long with light cycles ranging from 6am-6 pm and dark cycles ranging from 6 pm to 6 am. All mice were fed *ad libitum* with standard chow, and all metabolic data were analyzed using CalR software (https://calrapp.org).

### 2.8. Mouse motor and balance assessments: Grip strength, rotarod, and hindlimb clasping

Grip strength assesses neuromuscular function and maximal strength of forelimb and hindlimb muscles and was performed along with rotarod and hindlimb clasp assessments as indicated. Grip strength measurement involves holding the mouse by the tail and having it grip the top portion of the grid with its forelimbs and hindlimbs in three separate trials, recording the highest score from three different measurements, and not allowing any prior acclimation by the mice.

The rotarod was used to measure motor coordination and balance, performed at the indicated weeks before and after treatments and using week 5 as baseline measurement. The rotarod device (Ugo Basile) consisted of a revolving rod with five lanes, and assessment was performed over 3 days, each day consisting of an initial acclimation period during which mice were kept on the rotarod at a constant 5 rpm velocity for a total of 6 min. Acclimation was followed by three trials for each mouse with at least a 10-minute break between trials. During each trial the rod was set to accelerate from 0 to 50 rpm over a maximum span of 5 min, and the time a mouse falls from the rod was recorded.

Hindlimb clasping is often used to assess neurodegeneration in mouse HD models and is measured using a three point scale, with 0 reflecting no evident disease onset and 3 reflecting severe disease manifestation. Testing was carried out during the indicated weeks pre- and post-treatments. Prior to testing, mice were acclimated to the testing room for 30 min, followed by three trials each separated by 10 min resting intervals. The mouse was held by its tail for 10 s and its level of clasping recorded.

### 2.9. Statistics and bioinformatics

Technical and biological sample replicates and individual mouse measurements were recorded, plotted and analyzed by GraphPad Prism 7 (La Jolla, CA) software. Plots show single, median or mean values (+/− SD), statistically compared by two-tailed Student’s *t* test or one-way ANOVA F-test. Predicted linear regressions on plots (±95 % confidence intervals) used either Pearson (R_p_) or Spearman (R_s_) correlations. All statistical differences were considered significant if p ≤ 0.05. A hypergeometric distribution (Fisher’s exact test) was used to calculate the over-representation significance of differentially expressed genes in *M. musculus* Gene Ontology (GO) pathways. Pathways with a FDR-adjusted p-value ≤ 0.05 were considered significant. GO pathways were downloaded from the Mouse Genome Informatics repository (Law and Shaw, 2018). The package ComplexHeatmap (v.2.2.0) was used to visualize gene expression (Gu et al., 2016). Reactome pathway over-representation analysis was performed by inputing the significant differentially up- and down-regulated genes (Supplementary Table S1) into the public Reactome web browser package (version 73) (https://reactome.org/PathwayBrowser/). Downstream analyses were performed using Bioconductor R (www.bioconductor.org; v.3.6.3).

## 3. Results

Three sequential *in vivo* mouse studies were performed to: i) compare *N*-PPG induction of brain mitohormesis in HD and WT mice relative to vehicle (Veh) treated controls; ii) evaluate brain-controlled behavioral functions and metabolic responses in HD mice during *N*-PPG vs. Veh treatments; and iii) assess rebound brain and metabolic responses after extended *N*-PPG stress induced mitohormesis in healthy WT mice.

### 3.1. Short-term N-PPG induction of brain mitohormesis in HD and WT mice

**Fig. 1.**
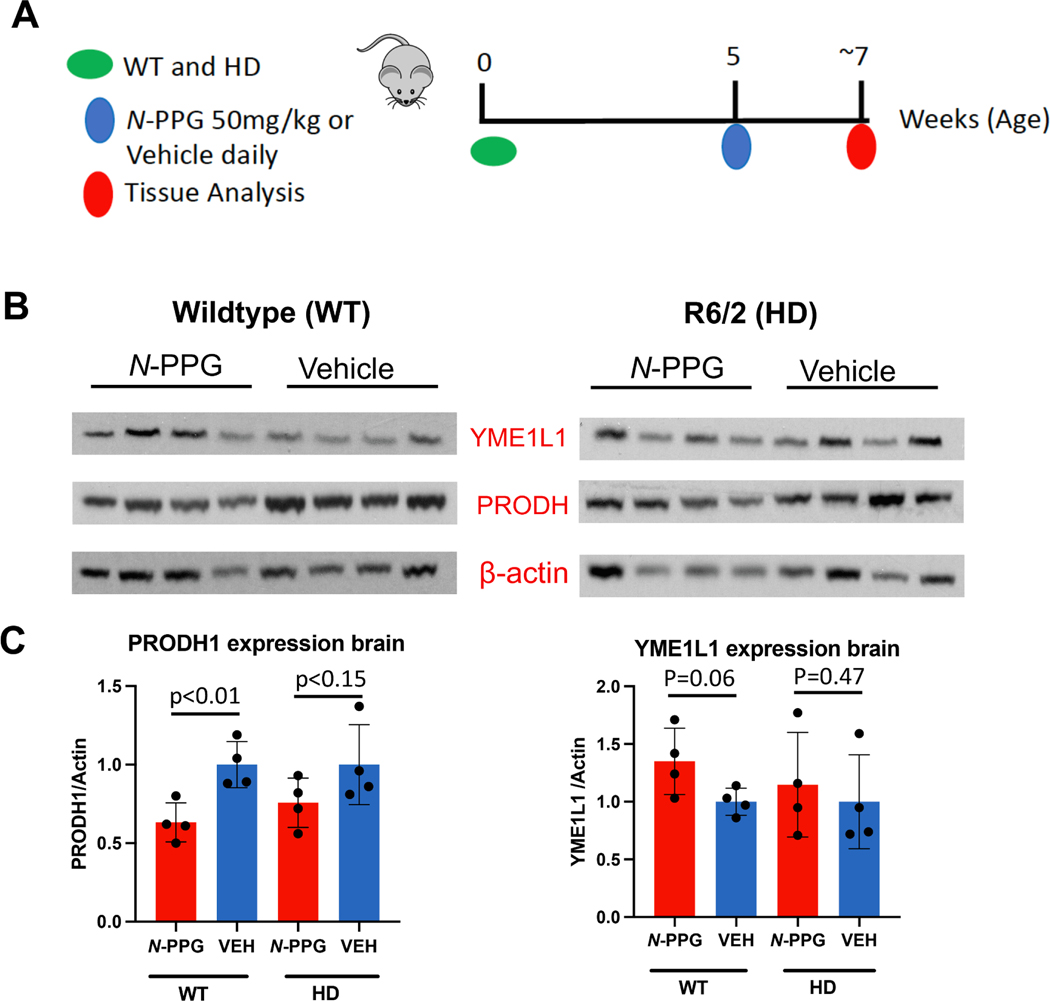
*N*-PPG induces brain mitohormesis in HD and WT mice. **A.** Schematic of study in which 5-week-old mice (R6/2 HD = 8, B6 WT = 8) receive daily gavage treatments (*N*-PPG at 50 mg/kg or Veh) for 9 days before sacrifice and snap freezing of whole brain and kidney tissue samples for later immunoblotting, RNA sequencing, and metabolomic analyses. **B.** Immunoblots of WT and HD whole brain samples probed for Prodh, Yme1l1, and β-actin with densitometry to determine treatment (*N*-PPG, Veh) induced Prodh/actin and Yme1l1/actin ratios. **C.** Quantification of the mean values (+/− SD) plotted in bar graphs; p values for treatment differences determined by ANOVA F testing.

We used the R6/2 (HD) mice that exhibit subcellular mitochondrial abnormalities (Creus-Muncunill and Ehrlich, 2019; Petersen et al., 2022) and develop early weight loss, enlarged cerebral ventricles, neuronal atrophy, progressive increase of mHTT aggregates, motor alterations and cognitive deficits to evaluate *N*-PPG. Randomized subgroups of WT (n = 8) and HD (n = 8) mice received daily oral gavages starting at age 5 weeks with either saline Veh or 50 mg/kg *N*-PPG, and within 2 h of their final dose (9th day, week 7) were sacrificed for resection and cryopreservation of kidney and whole brain tissue samples (Fig. 1A). In agreement with our previous study in WT mice (Scott et al., 2021), *N*-PPG also induced some evidence of mitohormesis in HD brain samples. Treated HD brains exhibited nominal (but insignificant) declines in Prodh protein levels associated with increases in Yme1l1 protease (Fig. 1B, 1C) and chaperone Grp78 (not shown) expression.

**Fig. 2.**
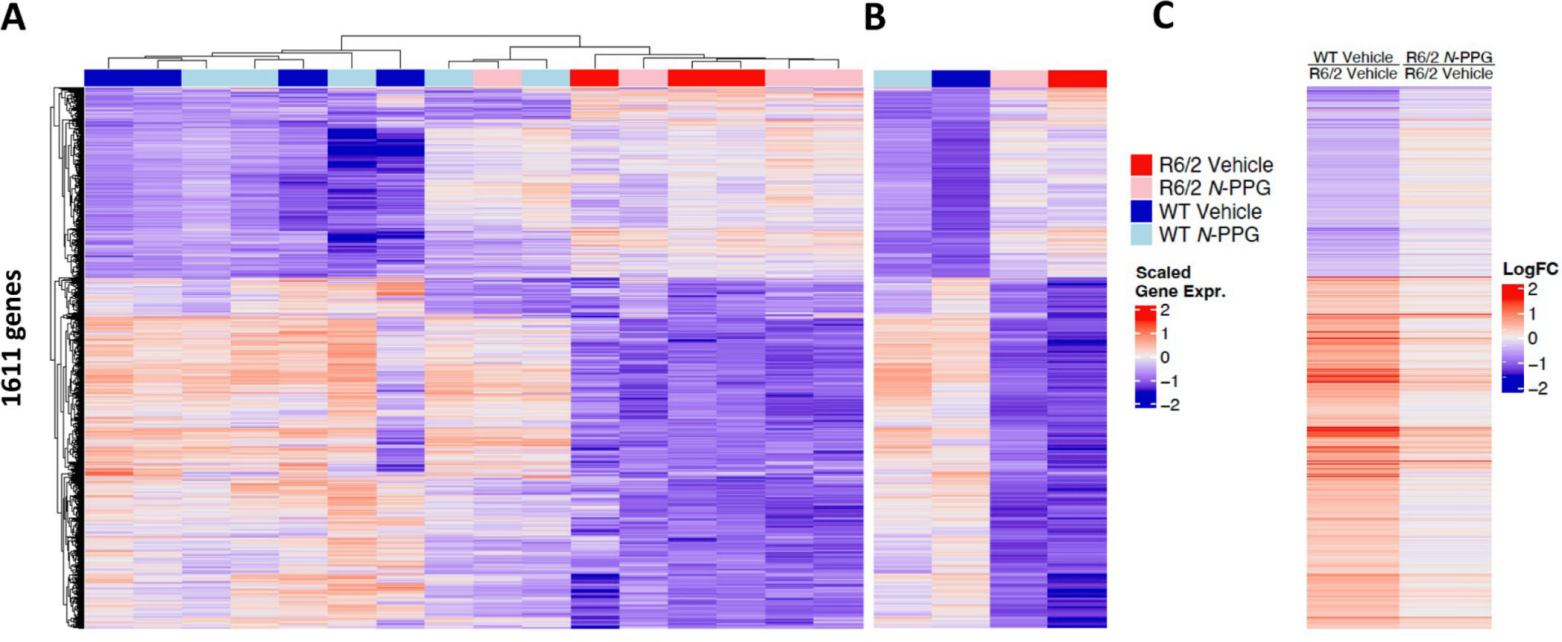
*N*-PPG treatment partially normalizes HD whole brain transcriptomes. **A.** Unsupervised clustering heat map of the RNAseq identified 1611 significant differentially expressed (SDE, FDR p < 0.05) genes (Log2 scaled) in whole brains of R6/2 HD and B6 WT mice, treated with *N*-PPG or Veh as described in Fig. 1. **B.** Heat map of four group mean gene expression values derived from panel A to illustrate overall transcriptome differences in WT and HD mice and to compare *N*-PPG treatment effects on HD and WT mouse brains. **C.** Heat map of mean gene ratios, derived from panel B scaled mean single gene values, to illustrate partial normalization of HD *N*-PPG/Veh gene ratios toward those of control (Veh) WT/HD gene ratios. Supplementary Table S1 provides numeric values and significance for all SDE gene expression and gene ratio changes shown on heat maps.

Total RNA was extracted from the WT (*N*-PPG = 4, Veh = 4) and HD (*N*-PPG = 4, Veh = 4) whole brain samples for RNA sequencing (RNAseq) that identified 1611 significant differentially expressed genes (SDE, with FDR p ≤ 0.05) between all samples (Fig. 2A, 2B; Suppl. Table S1). Interestingly, bioinformatic analyses revealed partial normalization by *N*-PPG of the HD whole brain transcriptomes toward those of control WT mice with respect to the SDE gene set (Fig. 2B, 2C), and identified one HD mouse that appeared by unsupervised clustering analysis more like treated WT mice than other treated or control HD mice (Fig. 2A). A strong linear Pearson correlation (R_p_ = 0.75) between control WT/HD SDE gene ratios and treated/control HD SDE gene ratios, consistent with the heat maps, also confirmed that short-term *N*-PPG treatment partially normalized HD brain transcriptomes (Suppl. Figure S1A; Suppl. Tables S2A, S2B). This pattern of whole brain SDE gene normalization included three significantly downregulated genes in the HD brains: tyrosine hydroxylase (*Th*), dopamine receptor-1 (*Drd1*), and adenosine A2A receptor (*Adora2a*). Average *Th* mRNA levels in untreated HD mice were upregulated by *N*-PPG to 40 % of control WT levels, average *Drd1* levels were upregulated to 44 %, and average *Adora2a* levels were upregulated to 32 % of control WT brain levels with the *N*-PPG treatment (Suppl. Figure S1B). GO analysis of the SDE genes confirmed downregulation of neural function in HD relative to WT brains, including significant downregulation of *Th* and *Drd1* in HD relative to WT brains, consistent with striatal-enriched gene expression changes reported in 6 month old R6/1 (HD) mice (Desplats et al., 2006). Likewise, molecular pathway normalization by *N*-PPG treatment was further evidenced by Gene Set Enrichment Analysis (GSEA). Rank ordering the most significantly altered (p < 0.01) GSEA pathway NES (normalized enrichment scores) values between *N*-PPG treated WT mouse brains relative to control WT brains, we identified the glutamatergic synapse pathway as the most positively up-regulated and the mitochondrial respiratory chain complex 1 assembly pathway as the most negatively down-regulated brain pathways in WT mice treated with *N*-PPG (Suppl. Figure S1C). To evaluate overall pathway normalization in HD mouse brains following *N*-PPG treatment, we looked at the set of 20,207 expressed mouse brain genes representing > 8500 different GSEA pathways expressed in all control and *N*-PPG treated WT and HD mouse brains. In support of pathway normalization, we found a statistical excess of overlapping GSEA pathways between *N*-PPG treated HD and vehicle treated (control) WT mouse brains. Specifically, when both groups were compared (relative to vehicle treated HD brains), we found 594 HD pathways significantly up-regulated (315) and down-regulated (279) by *N*-PPG treatment relative to 793 pathways significantly up-regulated (476) and down-regulated (317) by vehicle treatment. Among these overlapping pathways were a significant excess of 65 up-regulated (p < 8.82 e^−20^) and 23 down-regulated (p < 6.17 e^−4^) GSEA pathways (Suppl. Figure S1D). Included in the set of overlapping (normalized) upregulated GSEA pathways were glutamatergic synapse (for groups R6/2*N*-PPG vs. R6/2 Vehicle: NES = +1.27, p = 0.036; for groups WT Vehicle vs. R6/2 Vehicle: NES = +2.06, p = 0.002) and mitochondrial respiratory chain complex 1 assembly response (for groups R6/2*N*-PPG vs. R6/2 Vehicle: NES = +1.63, p = 0.029; for groups WT Vehicle vs. R6/2 Vehicle: NES = +1.82, p = 0.004).

### 3.2. Health, behavioral and metabolic impacts on HD mice treated with N-PPG

**Fig. 3.**
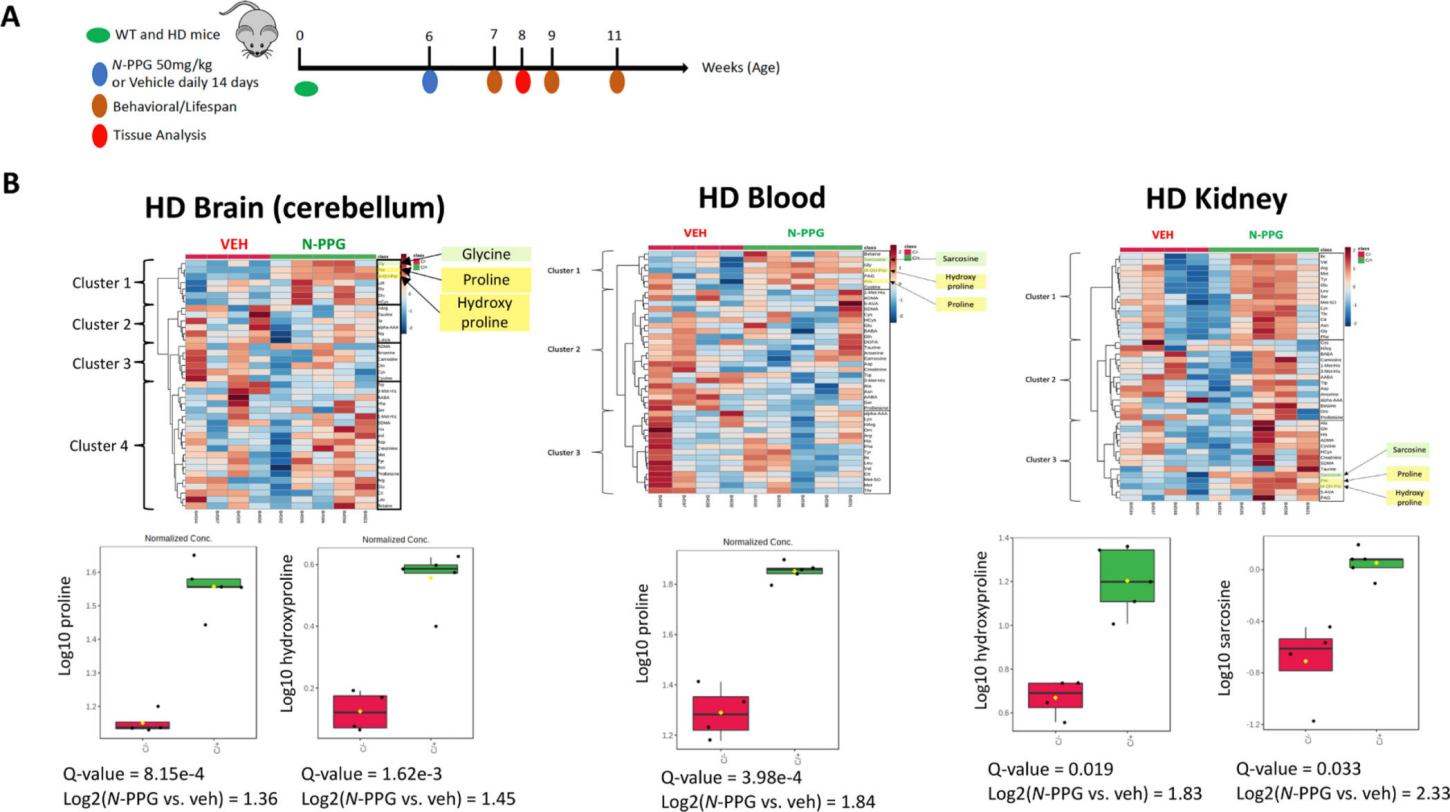
*N*-PPG treatment increases proline, hydroxyproline and sarcosine in HD mice. **A.** Schematic of study in which 6 week old HD mice received daily gavage treatments (*N*-PPG at 50 mg/kg or Veh) for 14 days before half were sacrificed for metabolomic profiling and the rest were followed for behavioral measurements and longevity. **B.** Metabolomic profiling of brain (cerebellum), blood and kidney samples collected at 14 days showed as displayed on the heat map the impact of *N*-PPG treatment on various clusters of quantified amino acid-like metabolites, including the smaller clade of proline, hydroxyproline and sarcosine. Box-whisker plots show the *N*-PPG induced group and average increases in brain proline (2.6-fold) and hydroxyproline (2.7-fold), blood proline (3.6-fold), and kidney hydroxyproline (3.6-fold) and sarcosine (5.0-fold), with Q-values indicating significant p-values adjusted for FDR.

In two prior studies using tumor-bearing nu/nu athymic mice (Scott et al., 2019) and C57BL/6J male and female WT mice (Scott et al., 2021), short-term oral dosing of 50–200 mg/kg *N*-PPG for up to 9 days produced no significant adverse health impacts as assessed by mouse weight and activity. Beyond these standard cage assessments, more in depth monitoring for potential adverse *N*-PPG treatment effects was undertaken in 6 week old HD (R6/2) mice, orally treated with Veh (n = 9) or 50 mg/kg *N*-PPG for 14 days (Fig. 3A). The mice were followed for behavioral and metabolic tests (grip strength, rotarod, hindlimb clasp, and metabolic cage parameters) and lifespan (Suppl. Figure S2). Overall lifespans of Veh and *N*-PPG treated HD mice were not significantly different (median survival = 14.3 weeks). Repeated weight and motor performance measurements (with exception of hindlimb clasp) of the HD mice declined as expected after age 9 weeks, with no significant differences observed in the two treatment arms (Suppl. Figure S2). Metabolic cage analyses at 12 weeks showed expected increased activity and metabolic parameters by all HD mice during night/dark cycles relative to day/light cycles; *N*-PPG treated HD mice showed non-significant trends toward more overall O_2_ consumption, CO_2_ production, food consumption and energy expenditure (Suppl. Figure S2).

In addition, HD mice treated for 14 days were sacrificed to collect blood, kidney and brain (cerebellum) samples for metabolomic analysis (Fig. 3A). More specifically, we sought to determine if *N*-PPG inhibition of mitochondrial Prodh produced any significant tissue changes in proline or other amino acid-related metabolites. Of the 536 analytes quantifiable across the different HD tissue samples by our metabolomic platform, 260 were common to all three tissue types, and 39 of these metabolites were classified as either amino acids or amino acid-related (Suppl. Figure S3; Suppl. Table S3). Supervised analysis of these metabolites identified a small *N*-PPG induced amino acid cluster containing proline and/or hydroxyproline, and in two of the tissue types (blood, kidney), this cluster also contained the glycine-related metabolite, sarcosine. Notably, the three co-clustered amino acid-related metabolites were most prominently increased in the *N*-PPG treated HD kidney samples (Fig. 3B). Average *N*-PPG induced cerebellar proline levels were increased 2.6-fold, and hydroxyproline levels were induced 2.7-fold. Average blood hydroxyproline levels were induced 3.6-fold and average kidney hydroxyproline levels were likewise induced 3.6-fold, whereas kidney sarcosine levels were increased 5.0-fold.

**Fig. 4.**
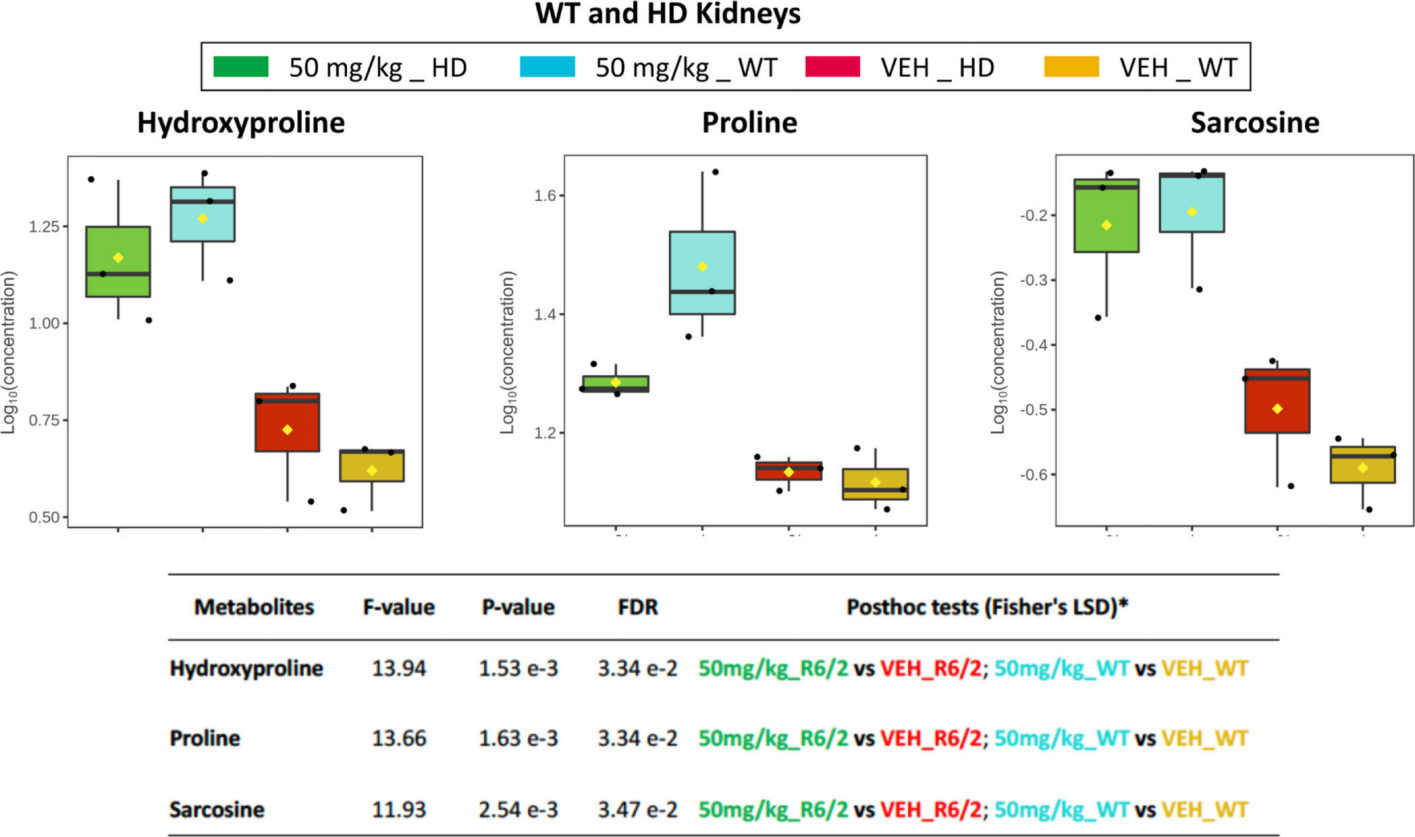
*N*-PPG increases proline, hydroxyproline and sarcosine in WT and HD kidneys. Kidney samples (n = 12) from the *N*-PPG- and Veh-treated mice described in Fig. 1A were profiled for proline, hydroxyproline and sarcosine. Box-whisker plots for the four different groups (n = 3 mice/group) show significant *N*-PPG induced increases in each of the three metabolites for both WT and HD mice, as shown by calculated Fisher’s LSD F, p, and FDR values. Of note, WT samples relative to treated HD samples showed consistently greater *N*-PPG inductions of proline (2.4- vs. 1.4-fold), hydroxyproline (4.6- vs. 2.8-fold), and sarcosine (2.5- vs. 1.9-fold).

To learn whether these HD kidney metabolome changes after *N*-PPG treatment differed from *N*-PPG induced changes in WT mouse kidneys, we similarly assayed amino acid-related changes in our earlier collected HD and WT kidney samples. Hierarchical clustering of the quantified 20 amino acids and 21 amino acid-related metabolites in the 12 different kidney samples (three mice per subgroups) again revealed the small cluster of proline, hydroxyproline, and sarcosine metabolites as induced by *N*-PPG treatment in both WT and HD kidney subgroups, but with consistently greater WT vs. HD inductions of proline (2.4-vs. 1.4-fold), hydroxyproline (4.6-vs. 2.8-fold), and sarcosine (2.5-vs. 1.9-fold) (Fig. 4; Suppl. Table S4).

**Fig. 5.**
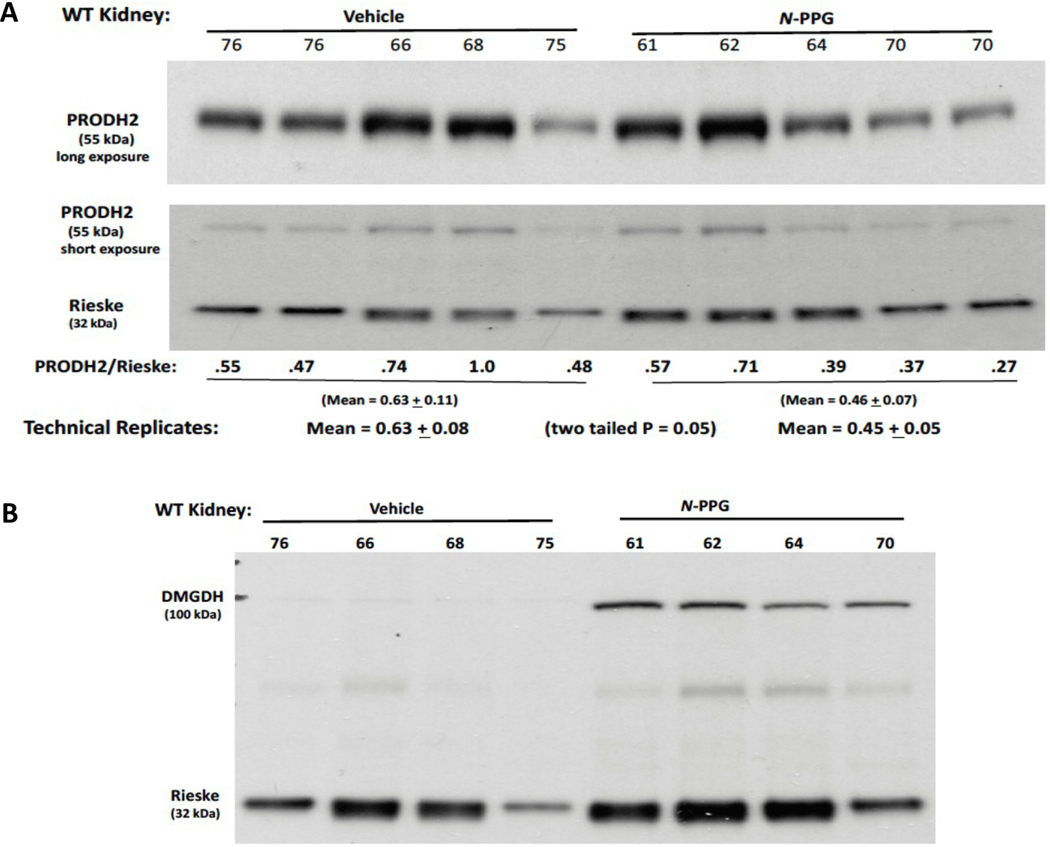
*N*-PPG decreases mitochondrial PRODH2/HYPDH but increases mitochondrial DMGDH. Protein lysates from WT Veh (n = 4) and *N*-PPG (n = 4) treated kidney samples (from Fig. 4) were immunblotted for two mitochondrial enzymes thought to control hydroxyproline (Prodh2/Hypdh) and sarcosine (Dmgdh) levels. **A.** *N*-PPG caused a mean 30 % reduction (p = 0.05) in Prodh2 (long exposure)/Rieske (short exposure) levels. **B.** Using the same protein lysates, *N*-PPG caused a dramatic (>10-fold) increase in Dmgdh expression.

To understand these unexpected *N*-PPG inductions of hydroxyproline and sarcosine, the residual WT kidney samples were probed for protein changes in kidney enzymes potentially responsible for controlling hydroxyproline (mitochondrial Prodh2/Hypdh) and sarcosine (mitochondrial Dmgdh and Sardh, cytosolic Gnmt) levels. Hydroxyproline is exclusively catabolized by Prodh2, a paralog with structural similarity to Prodh but expressed primarily in kidney and liver mitochondria. Thus, we surmised that if *N*-PPG acts as a similar suicide inhibitor of Prodh2, then *N*-PPG treated kidney Prodh2 levels might similarly decline in conjunction with Prodh. Immunoblotting the kidney lysates confirmed a 30 % reduction in Prodh2 with *N*-PPG treatment (Fig. 5A), comparable to the previously measured Prodh decline (Fig. 1, B and C). The synthesis of sarcosine, not detected in any of our brain samples but prominent in all our kidney samples, is known to be regulated by mitochondrial dimethylglycine dehydrogenase (Dmgdh) and cytosolic glycine N-methyltransferase (Gnmt), and its catabolism by conversion to glycine via sarcosine dehydrogenase (Sardh) (Palmieri et al., 2022). Our *N*-PPG treated WT kidney lysates revealed a modest increase in Sardh protein levels overshadowed by a dramatic increase (>10-fold) in DMGDH protein levels normalized to mitochondrial Rieske (Fig. 5).

### 3.3. Understanding brain rebound responses after extended mitohormesis induction in WT mice

**Fig. 6.**
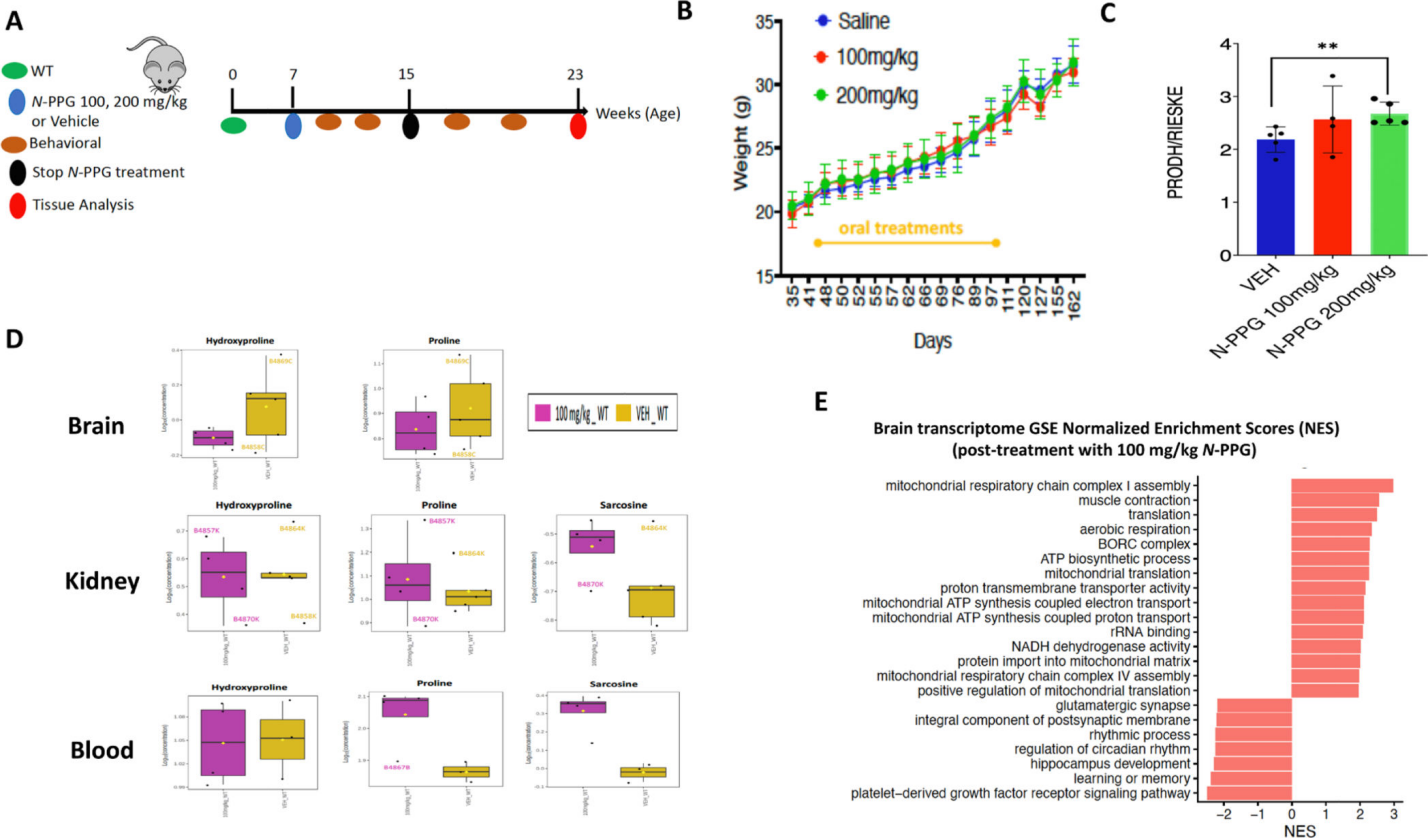
Rebound brain and metabolic responses after extended *N*-PPG treatment of WT mice. **A.** Schematic of study in which 7-week-old WT mice received extended daily gavage treatments of Veh (n = 5) or *N*-PPG at doses of 100 mg/kg (n = 5) or 200 mg/kg (n = 6) for 8 weeks, followed by 8 weeks off all treatments before sacrifice and tissue collection (brain, blood, kidneys). **B.** Body weights during and after treatment demonstrate no significant treatment effect. **C.** Whole brain immunoblot measured densitometric ratios showed significant (p < 0.01) *N*-PPG induced mitochondrial rebound of Prodh/Rieske expression above normal (Veh). **D.** Metabolomic profiling of the post-treatment (100 mg/kg *N*-PPG) WT brain (cerebellum), kidney and blood samples showed no significant changes in brain or kidney proline or hydroxyproline levels, a nominal (but not significant) increase in kidney sarcosine levels, but persistent significant increases in blood proline and sarcosine levels. **E.** RNAseq of post-treatment Veh (n = 4) and *N*-PPG (100 mg/kg, n = 4) WT brain/striatum samples were analyzed by GSEA for significant NES showed that 10 of the 15 highest expressed (NES≥ +2, p < 0.01) GO enrichment terms in the post *N*-PPG treated samples specifically involved mitochondrial pathways, including *mitochondrial respiratory chain complex 1 assembly* (NES = +3, p < 0.01).

We next questioned if any durable stress rebound responses might occur after 2 months of higher *N*-PPG dosing (>50 mg/kg per day) and then 2 months of therapy cessation (Fig. 6A). To test for stress rebound, 7 week old WT mice were randomized to receive daily oral gavage of 100 or 200 mg/kg of *N*-PPG or saline (Veh) for 8 weeks, at which time they were subjected to behavioral and metabolic cage testing. WT mice were then followed for an additional 8 weeks off all treatments, retested as before and then sacrificed to assess brain, blood and kidney samples for potential rebound responses to *N*-PPG stress-induced mitohormesis. No differences were noted in Veh and *N*-PPG treated WT mice during the first 8 weeks on therapy or the 8 week interval after therapy cessation with regard to weight change, motor activity or memory (Fig. 6B). However, relative to HD mice treated with the 50 mg/kg dose of *N*-PPG, WT mice treated with the higher doses of *N*-PPG showed greater average energy expenditure, oxygen consumption and carbon dioxide production than control WT mice while on treatment. By 2 months after treatment cessation, these metabolic patterns had completely reversed (Suppl. Figure S4). WT whole brain samples taken 8 weeks after cessation of *N*-PPG treatment showed increases in mitochondrial Prodh expression levels normalized to Rieske (Fig. 6C), indicating a mitohormesis rebound above the normal Prodh levels of control WT mice, in opposition to the declines in mitochondrial Prodh measured during 50 mg/kg *N*-PPG treatment (Fig. 1, B and C) or in our earlier study of mice treated with 50–200 mg/kg *N*-PPG (Scott et al., 2021).

Metabolomic profiling of the post-treatment WT brain (cerebellum), kidney and blood tissues showed rebound changes in amino acid-related metabolites by unsupervised hierarchical clustering, but only in the blood samples (Suppl. Figure S5); these included persistent increases in blood proline and sarcosine levels (Fig. 6D). Post-treatment kidney samples showed nominal (but not significant) increases in sarcosine levels relative to post-treatment control WT mice (Suppl. Table S5). The post-treatment cerebellar brain samples showed nominal (but not significant) decreases in proline and hydroxyproline levels, potentially related to the rebound increases in whole brain Prodh expression (Fig. 6D).

**Fig. 7.**
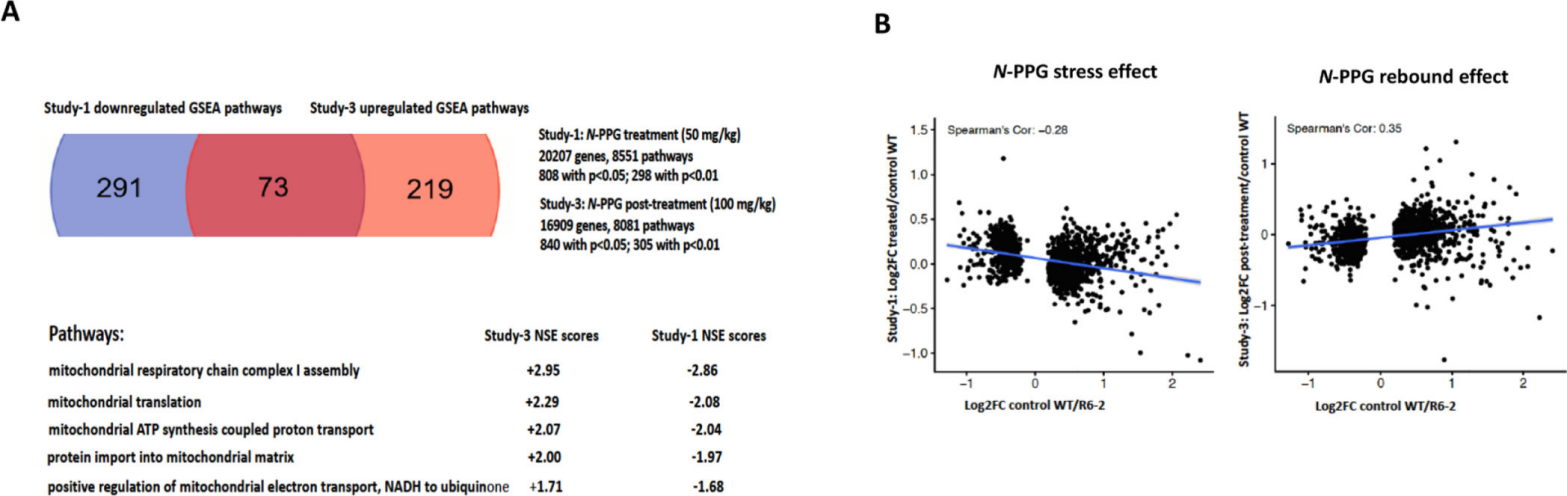
*N*-PPG post-treatment rebounds in brain transcriptome and mitochondrial pathways. **A.** Comparison of GSEA pathways significantly downregulated in WT mouse brains treated with 50 mg/kg *N*-PPG (from Fig. 2 study) with those significantly upregulated in WT mouse brains post-treatment with 100 mg/kg *N*-PPG (from Fig. 6 study) identified 73 overlapping pathways with inverse NES scores including five mitochondrial pathways, led by *mitochondrial respiratory chain complex 1 assembly* with NES = −2.86 during *N*-PPG treatment rebounding to NES = +2.95 following *N*-PPG treatment. **B.** The whole brain 1611 SDE gene set identified during 50 mg/kg *N*-PPG treatment (from Fig. 2 study) were used to produce control (Veh) WT/HD gene ratios for correlation with either *N*-PPG treated (50 mg/kg) or *N*-PPG post-treatment (100 mg/kg) WT/Veh WT gene ratios, yielding inverse linear regressions and Spearman correlations (−0.28 and + 0.35, respectively).

Two months post-treatment brain (striatum) transcriptomic rebound changes were assessed in samples from control and 100 mg/kg *N*-PPG treated WT mice. Transcriptional changes were analyzed by GSEA for specific enrichment of mitochondrial pathways potentially signifying a durable brain mitohormesis rebound response after the 2 month induction of mitochondrial stress by *N*-PPG. We found 305 (p < 0.01) up- or down-regulated genes between the vehicle and *N*-PPG treated groups. While four of the six lowest expressed (NES ≤ −2, p < 0.01) GO enrichment terms in the post-treated brain/striatum samples specifically involved neurologic pathways, none included mitochondrial pathways. In contrast, ten of the fifteen highest expressed (NES≥ +2, p < 0.01) GO enrichment terms in the same samples specifically involved mitochondrial pathways (Fig. 6E), with the highest up-regulated pathway durably induced after *N*-PPG (relative to vehicle) cessation being *mitochondrial respiratory chain complex 1 assembly* (NES= +3, p < 0.01), exceeding the significant enrichment of this same pathway down-regulated during *N*-PPG treatment in our whole brain samples. A comparison of the significantly altered GO mitochondrial pathways down-regulated in WT whole brain samples during *N*-PPG treatment (50 mg/kg), with up-regulated mitochondrial pathways in rebounding WT striatal brain 2 months after cessation of *N*-PPG treatment (100 mg/kg), demonstrated a strikingly inverse enrichment in the top five pathway NES scores down-regulated during *N*-PPG treatment and up-regulation after treatment cessation (Fig. 7A). This inverse relationship was also apparent when the entire profile of 1611 SDE genes during treatment was compared with that after treatment cessation, with respect to gene ratios (Log2FC treated/control WT during treatment and Log2FC post-treated/post-control WT) plotted against the same range of Log2FC control WT/HD ratios. These showed Spearman linear regression coefficients (R_S_) switching from −0.26 to + 0.35 (Fig. 7B, Suppl. Table S6), reflecting rebound of the post-*N*-PPG treated mouse brain transcriptomes from their expression patterns during *N*-PPG treatment stress.

## 4. Discussion

### 4.1. N-PPG triggers UPR^mt^ and mitohormesis in R6/2 mice, partially normalizing their brain transcriptomes

HD mice expressing the R6/2 exon-1 mHTT protein with ~ 150 polyQ repeats exhibit aggressive and early symptomatology followed by rapid demise (Li et al., 2005). Since mHTT is known to induce UPR^mt^ (Wang et al., 2022), it was uncertain whether or not *N*-PPG could trigger UPR^mt^ and brain mitohormesis in this aggressive HD mouse model. However, *N*-PPG treated R6/2 mice clearly demonstrated whole brain induction of UPR^mt^ and mitohormesis comparable to that in young WT mice, characterized by decay in the mitochondrial Prodh protein along with increased expression of mitochondrial chaperone and Yme1l1 protease proteins.

Our comparative whole brain transcriptome (RNAseq) analyses of R6/2 and WT mouse brains were consistent with the marked brain transcriptome changes reported in untreated R6/2 mice (Tang et al., 2011; Huang et al., 2022). More surprising, however, and despite overlap between whole brain transcriptomes of Veh and *N*-PPG treated WT and R6/2 mice limited to only 1611 SDE genes, bioinformatic analyses revealed partial normalization of this gene set in R6/2 mice toward that of normal WT mice, confirmed by highly correlated (R_p_ = 0.75) gene ratios. Also validated by GO analysis, this pattern of whole brain transcriptome normalization included three specific transcripts known to be dysregulated during HD disease progression and significantly downregulated in our Veh treated 7 week old R6/2 mice relative to WT mice: *Th*, *Drd1*, and *Adora2a*. In looking for brain pathway changes commonly altered by *N*-PPG in both WT and R6/2 mice, GSEA identified the *glutamatergic synapse* pathway as stimulated and the *mitochondrial chain complex 1 assembly* pathway as down-regulated. Remarkably, the latter mitochondrial pathway repressed during *N*-PPG treatment emerged by transcriptome and GSEA analysis as the most strongly to rebound above normal months after cessation of extended *N*-PPG treatment.

### 4.2. R6/2 mice had no ill effects from N-PPG treatment but, along with WT mice, showed unexpected metabolic changes

Prior studies in otherwise healthy mice failed to reveal any systemic ill effects from short term treatment with *N*-PPG doses ranging from 50 to 200 mg/kg (Scott et al., 2019; Scott et al., 2021). However, this study was undertaken to perform more in depth monitoring of its health effects in neurologically compromised R6/2 mice to predict the potential tolerability of *N*-PPG in HD-afflicted patients. Two weeks of oral *N*-PPG treatment (50 mg/kg) just before the onset of motor deficits and weight loss in R6/2 mice did not significantly affect their weights, motor or metabolic tests, or 14.3 week median survival, portending excellent *N*-PPG tolerability in future preclinical tests across various neurodegenerative disease models.

We performed comprehensive metabolomic profiling on blood, kidney and brain/cerebellum samples. We found that *N*-PPG brain penetration is sufficient to inhibit proline catabolism and increase brain proline levels 2.6-fold. Proline levels were nominally but not significantly elevated in the R6/2 treated blood and kidney samples. However, hydroxyproline levels were significantly elevated in all three tissue types (2.7- to 3.6-fold), and *N*-PPG significantly increased kidney levels of the glycine-related metabolite sarcosine. In view of these unexpected increases in hydroxyproline and sarcosine, we evaluated kidneys and found that *N*-PPG treatment produces comparable increases in proline, hydroxyproline and sarcosine in both WT and R6/2 mouse kidneys, with the former showing somewhat greater inductions of these three metabolites (Fig. 4).

To explain the unexpected tissue findings of comparably increased hydroxyproline and sarcosine levels in treated R6/2 and WT mice, we looked for other mitochondrial enzyme changes in WT kidney samples. We reasoned that *N*-PPG might increase hydroxyproline levels by its ability to act as a suicide inhibitor of the Prodh paralog, Prodh2/Hypdh, primarily expressed only in kidney and liver mitochondria. We noted a significant reduction in *N*-PPG treated kidney levels of Prodh2 in keeping with its similar mitohormesis-inducing decay in Prodh protein levels. Likewise, exploring multiple sarcosine regulating enzymes, we observed that *N*-PPG significantly increased mitochondrial levels of the mitochondrial sarcosine synthesizing enzyme, Dmgdh, in concert with the induced decay in Prodh. Thus, while we confirmed that *N*-PPG achieves sufficient brain concentrations to elevate brain proline levels in treated WT and R6/2 mice, more preclinical studies are needed to better understand the systemwide pharmacodynamic mechanisms and clinical implications of our unexpected finding that *N*-PPG increases hydroxyproline and sarcosine levels, as well as proline levels, in WT and R6/2 mouse tissues.

### 4.3. Brain and metabolic responses rebound after extended N-PPG treatment of WT mice, bringing new insights to the therapeutic application of mitohormesis induction

While new mitochondrial fitness strategies, including pharmacologic induction of CNS mitohormesis, have been called for to treat HD and other neurodegenerative diseases (Fu et al., 2019; Hu et al., 2021; Stoker et al., 2022), it remains unclear how best to employ any candidate mitohormesis-inducing agent such as *N*-PPG or to choose an appropriate neurodegenerative disease mouse model to evaluate its efficacy (Creus-Muncunill and Ehrlich, 2019; Hu et al., 2021). Thus, we looked for evidence of sustained CNS mitohormesis and/or metabolic rebound responses in WT mice completing a 2 month course of higher dose (100 or 200 mg/kg) *N*-PPG treatment. Two months after cessation of the well tolerated higher doses of *N*-PPG, the healthy post-treatment WT mice showed robust rebound increases in their whole brain mitochondrial Prodh protein levels. Metabolomic profiling of their cerebellar, kidney and blood samples also showed persistent increases in brain proline and hydroxyproline levels associated with increased kidney sarcosine and blood sarcosine and proline levels. We cannot readily explain these persistently elevated metabolite levels, especially given that unsupervised clustering of all the differentially expressed metabolites showed significant increases in more than 20 other metabolites restricted to the post-treatment blood samples. These findings suggest a complex systemwide metabolic rebound response to the earlier *N*-PPG treatment manifested primarily in blood levels. It would be of particular interest to learn if *N*-PPG induced hyperprolinemia and/or hyperhydroxyprolinemia can directly or indirectly impact either the structure or activity of other key metabolic proteins or even HTT itself (Phang, 2023).

Transcriptome analysis of the 100 mg/kg post-treatment brain/striatum samples revealed significant rebound increases in many genes and mitochondrial pathways shown to be depressed during 50 mg/kg *N*-PPG treatment. We compared the on-treatment and post-treatment transcriptome responses by plotting their common sets of 1611 SDE gene ratios against the same set of control gene ratios. This linear correlation yielded a Spearman coefficient of −0.28 for the 50 mg/kg *N*-PPG treatment samples that reversed to + 0.35 for the 100 mg/kg *N*-PPG post-treatment samples, signifying a general brain transcriptome rebound. We then compared the GSE identified mitochondrial pathways with highest (positive NES values) brain scores in the 100 mg/kg *N*-PPG post-treatment samples to the most reduced mitochondrial pathway scores (negative NES values) in the 50 mg/kg *N*-PPG treatment samples. This comparison yielded five common mitochondrial pathways each with reciprocal scores of comparable magnitude, led by the *mitochondrial chain complex 1 assembly* pathway. Apart from signifying a robustly rebounding mitochondrial transcriptome, as might be expected after treatment with a mitohormesis-inducing agent, this specific mitochondrial rebound response mechanistically reflects *N*-PPG’s ability to target and irreversibly inhibit the inner mitochondrial membrane enzyme, Prodh, whose normal cellular function is to support mitochondrial respiration by supplying anaplerotic glutamate and transferring electrons into the electron transport chain for ATP generation (Scott et al., 2019; Scott et al., 2021).

Although extended oral treatment with *N*-PPG at daily doses up to 200 mg/kg is exceptionally well tolerated in WT and R6/2 mice, daily treatment with 50 mg/kg *N*-PPG for 2 weeks does not appear to slow disease progression or extend the very short lifespan of R6/2 mice. Thus, a pharmacologic inducer of CNS mitohormesis should not be expected to reverse established neuropathology but, if given early enough and before irreversible mitochondrial dysfunction occurs (Hu et al., 2021), might prevent further disease progression. Our observed rebound in the brain mitochondrial responses of WT mice two months after *N*-PPG treatment cessation would appear to satisfy recent calls for a new pharmacologic candidate capable of enhancing mitochondrial fitness by inducing mitohormesis (Hu et al., 2021, Stoker et al., 2022). Based on our present mouse study results, we believe that future preclinical development and evaluation of a mitohormesis-inducing agent like *N*-PPG should employ a slower progressing HD (or other neurodegenerative disease) mouse model allowing for treatment early enough to induce a strong CNS mitohormesis response followed by treatment cessation and subsequent robust rebound in CNS mitochondrial pathways and fitness.

## 5. Conclusion

Our present findings in both WT and R6/2 mice point to the brain-penetrating and mitohormesis-inducing potential of the Prodh suicide inhibitor, *N*-PPG, and provide new rationale and application insights compelling its further preclinical evaluation in other models of human neurodegenerative diseases characterized by loss of mitochondrial proteostasis.

## Supplementary Material

MMC6

MMC8

MMC7

MMC5

MMC4

MMC3

MMC2

MMC1

## Data Availability

All datasets presented or described in this study can be found in supplementary figures and tables associated with this article.
