# Supplementary material for: Brain transcriptomic, metabolic and mitohormesis properties associated with N-propargylglycine treatment: A prevention strategy against neurodegeneration
Source: Brain Res. Author manuscript; Available in PMC 2024 Jul 28. (PMC11283822; doi:10.1016/j.brainres.2023.148733)

Supplementary Figure Legends

Supplementary Figure S1. Additional evidence from Figure 2 supporting the conclusion that *N*-PPG (50 mg/kg x 9 days) induced partial normalization of HD brain transcriptomes and pathways toward WT. **A.** Pearson's linear correlation ($R_P = +0.75$, $p<0.05$) between Log2-fold changes (Log2FC) in treated HD/control HT (HT/H) and control WT/HD (W/H) mean gene ratios (n=1611 SDE gene set, Suppl. Table S1). **B.** Normalization of set of 1611 SDE mean gene ratios with inset of heat map showing extent of normalization for three mouse brain transcripts (Th, Drd1, Adora2a) known to be dysregulated during HD disease progression and significantly downregulated in Veh treated R6/2 mice relative to Veh treated WT mice. **C.** Gene set enrichment analysis (GSEA) showing normalized enrichment scores (NES) from transcriptomes of *N*-PPG vs. vehicle treated WT mouse brains. **D.** Statistical excess of overlapping GSEA pathways (up- and down-regulated) indicating pathway normalization in *N*-PPG treated HD (R6/2) mouse brains relative toward those of WT mouse brains.

Supplementary Figure S2. Repeated weight, motor performance and survival measurements of HD R6/2 mice orally gavaged with either Veh (n=9) or 50 mg/kg *N*-PPG (n=8) for 14 days (also schematically shown in Figure 3A). A. No significant change in body weights during or after Veh or *N*-PPG treatments. **B.** No significant change in median 14.3 weeks of survival after Veh or *N*-PPG treatments. **C.** No significant change in metabolic cage measurement at 12 weeks of overall O_2 consumption, CO_2 production, food consumption or energy expenditure. **D.** No significant change in grip strength, rotarod coordination or hindlimb clasp measured at indicated time points during and after Veh or *N*-PPG treatments.

Supplementary Figure S3. Breakdown of all metabolites commonly measured in tissues (blood, brain/cerebellum, kidney) of Veh and *N*-PPG treated HD R6/2 mice, as schematically shown in Figure 3A. Of the 536 tissue analytes quantifiable by our metabolomic platform, 260 were common to all three tissue types; the 39 classified as either amino acids or amino acid-related metabolites were the subject of further analysis (Figure 3B, Suppl. Table S3).

Supplementary Figure S4. Differential metabolic cage effects on WT mice during and after *N*-PPG treatments (Veh, 100 mg/kg, or 200 mg/kg daily x 8 weeks followed by 8 weeks off all treatments), schematically shown in Figure 6A. During treatment cage measurements showed significant *N*-PPG induced increases in CO_2 production, O_2 consumption, and energy expenditure (kcal/h), which largely reversed by 8 weeks after treatment cessation.

Supplementary Figure S5. Unsupervised clustering of the amino acid-related metabolites measured in blood, brain (cerebellum) and kidney tissues of WT mice collected 8 weeks after Veh or *N*-PPG (100 mg/kg) treatment, schematically shown in Figure 6A. Only the blood samples showed significant changes in proline, sarcosine and 20 other amino acid-related metabolites, suggesting a complex systemwide rebound response to the earlier *N*-PPG treatment.

Suppl. Figure S1

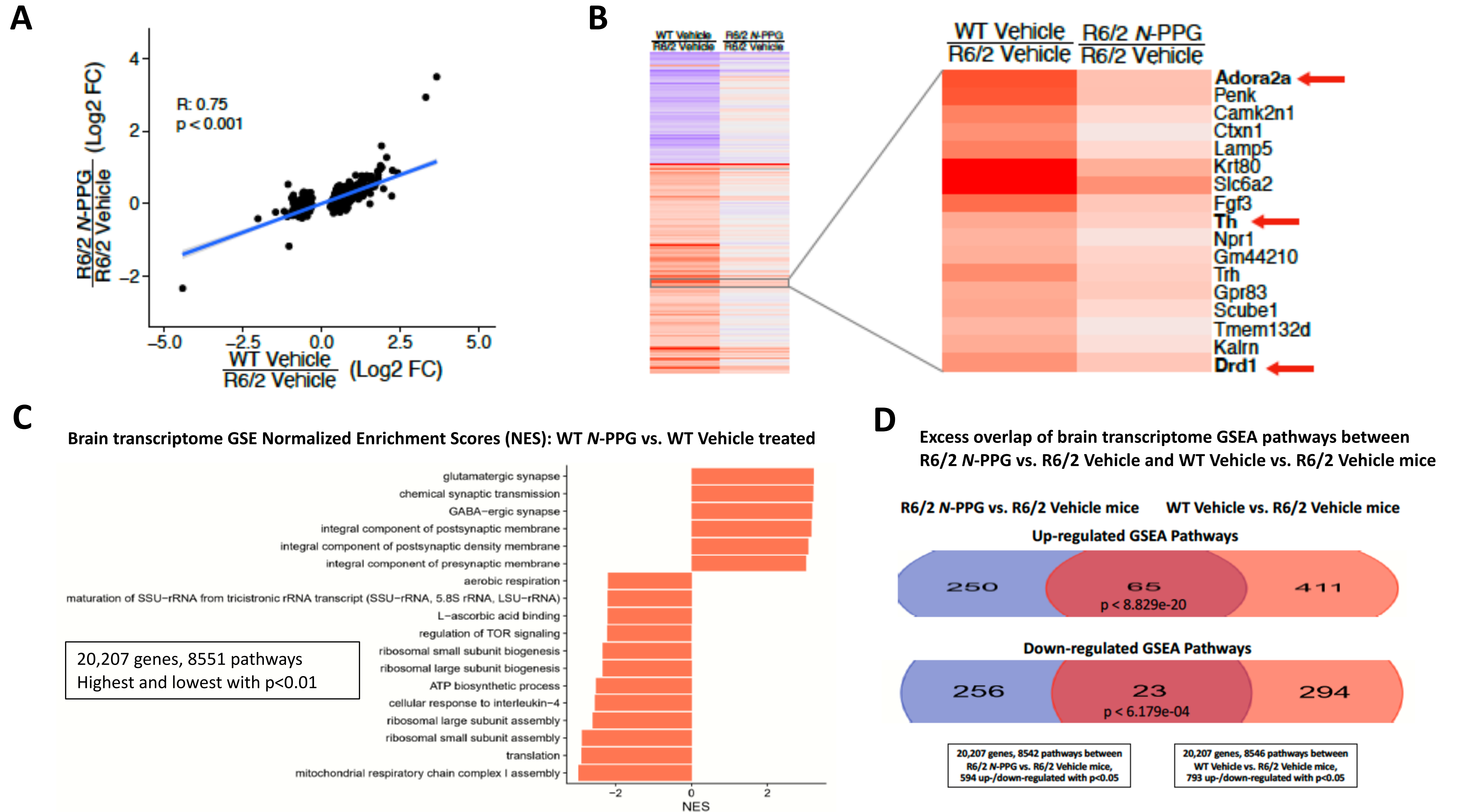

Suppl. Figure S2

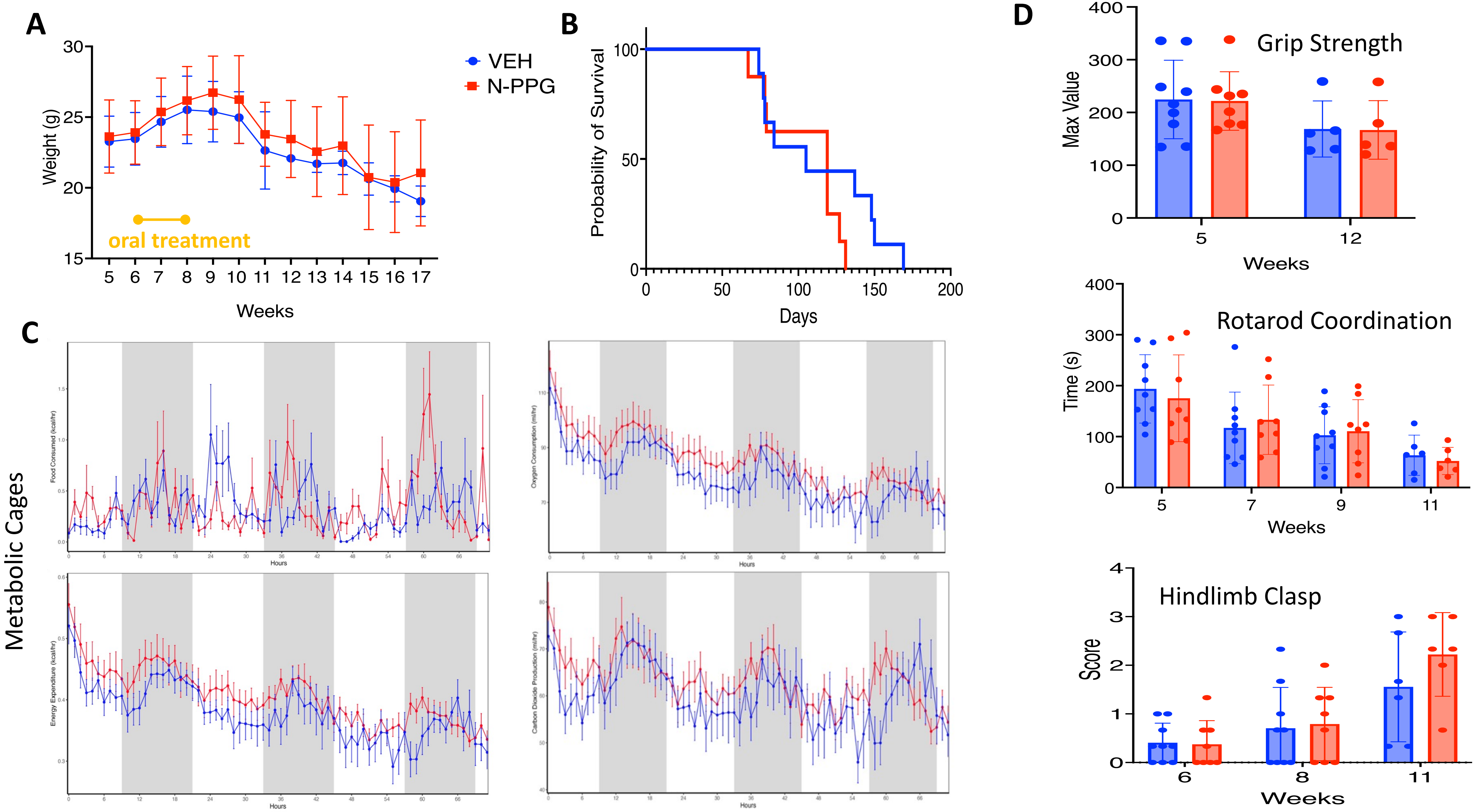

Suppl. Figure S3

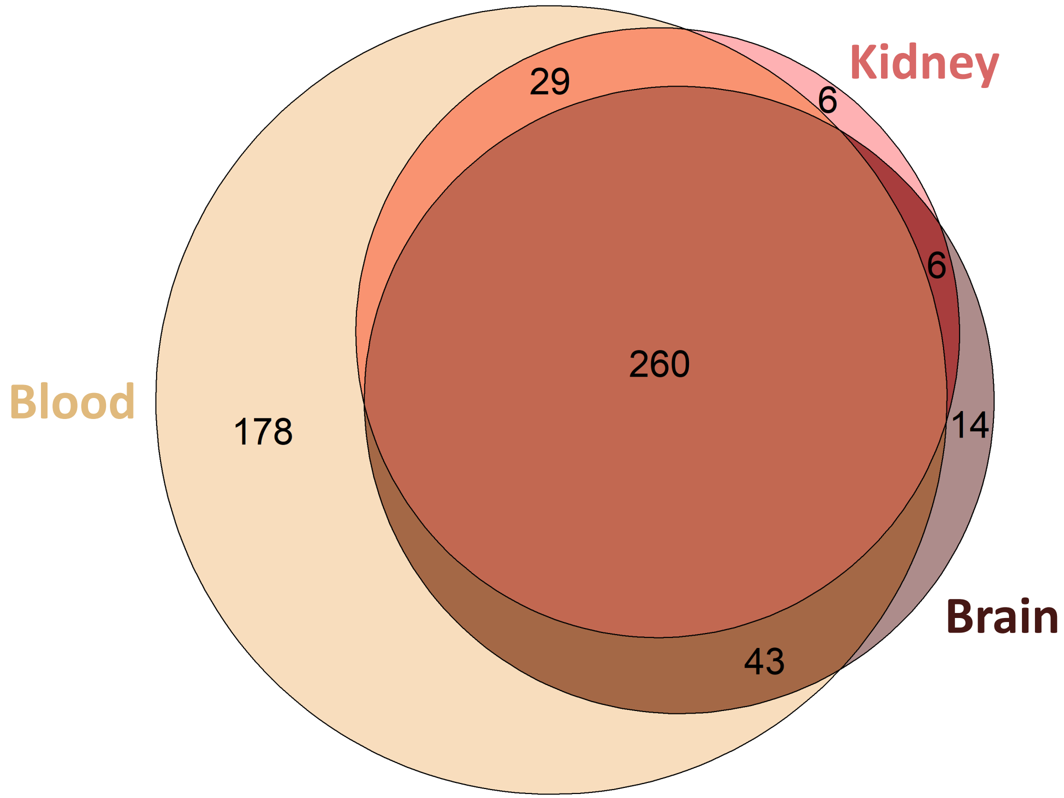

260 common to blood, brain and kidney

Class	Number of metabolites
Acylcarnitines	37
Alkaloids	1
Aminoacids	20
Aminoacids Related	19
Bile Acids	1
Biogenic Amines	6
Carboxylic Acids	1
Ceramides	14
Cholesterol Esters	2
Diacylglycerols	6
Fatty Acids	3
Glycerophospholipids	81
Glycosylceramides	11
Indoles Derivatives	1
Nucleobases Related	2
Sphingolipids	14
Sugars	1
Triacylglycerols	39
Vitamins & Cofactors	1

39 common to blood, brain and kidney

Amino acids (20)	Amino acid-related (19)
Ala	1-Met-His
Arg	3-Met-His
Asn	5-AVA
Asp	AABA
Cys	ADMA
Gln	alpha-AAA
Glu	Anserine
Gly	Betaine
His	Carnosine
Ile	Cit
Leu	Creatinine
Lys	Cystine
Met	HArg
Phe	HCys
Pro	Orn
Ser	ProBetaine
Thr	SDMA
Trp	t4-OH-Pro
Tyr	Taurine
Val	

Suppl. Figure S4

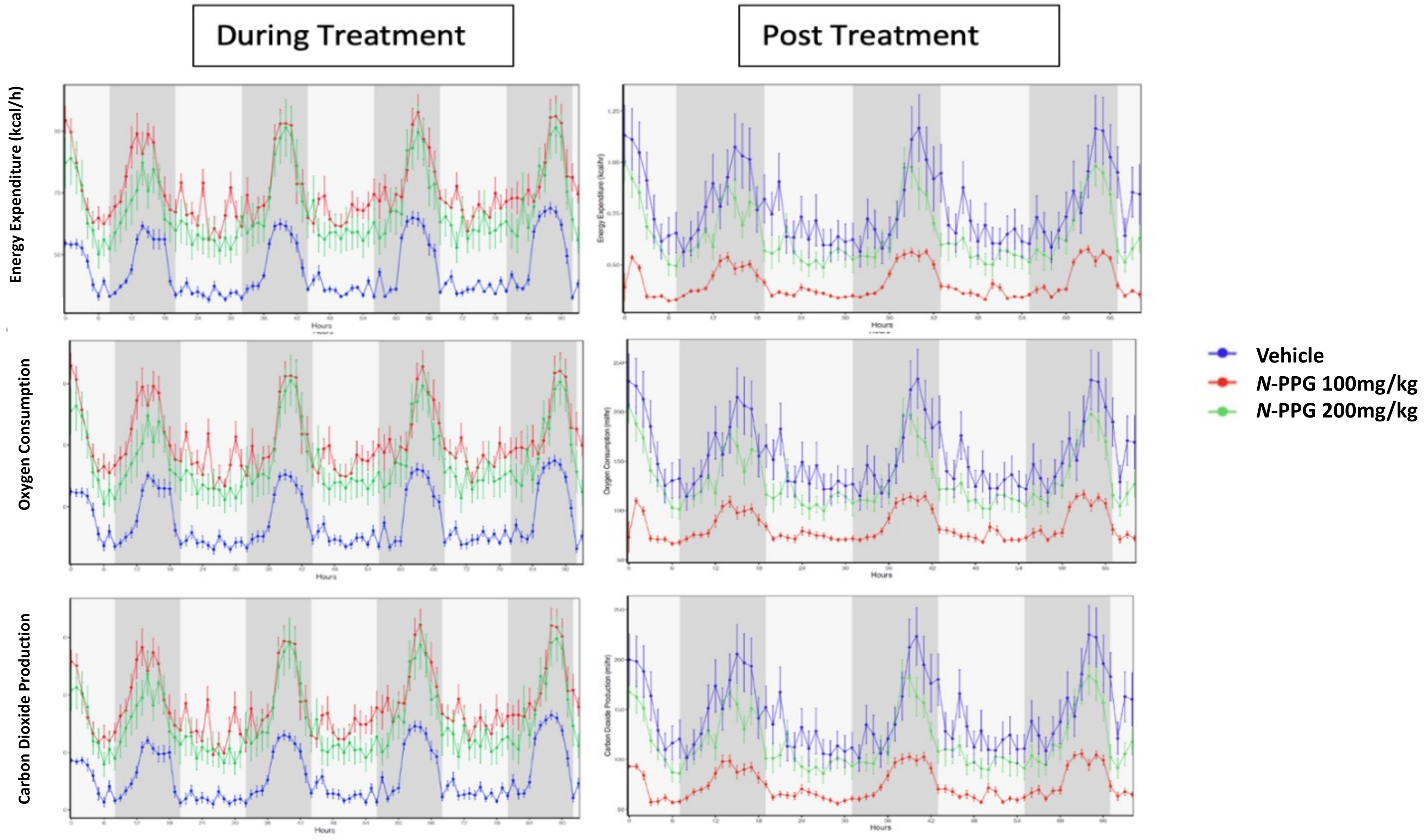

Suppl. Figure S5

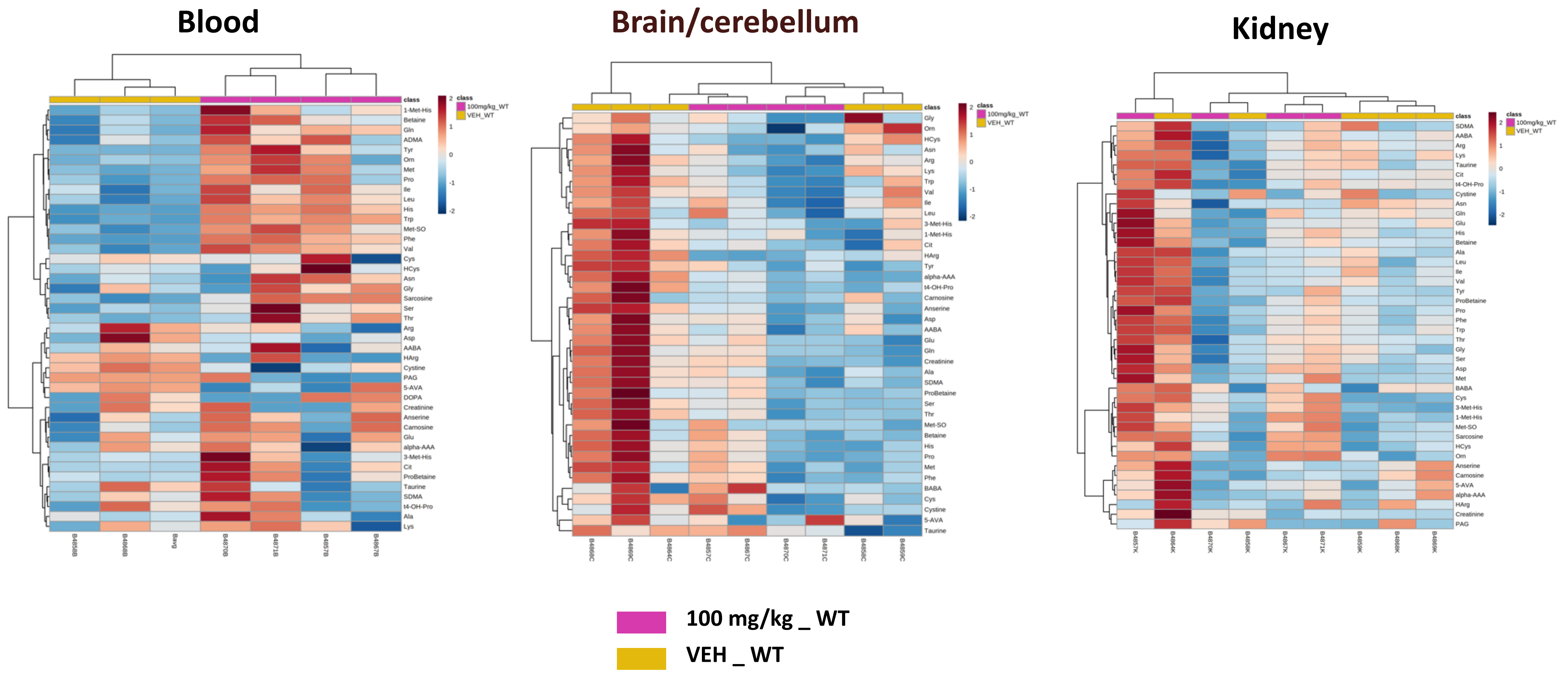

Supplement: MMC1 [file NIHMS1958532-supplement-MMC1.pdf]
